# Coronary heart disease and risk for cognitive impairment or dementia: Systematic review and meta-analysis

**DOI:** 10.1371/journal.pone.0184244

**Published:** 2017-09-08

**Authors:** Kay Deckers, Syenna H. J. Schievink, Maria M. F. Rodriquez, Robert J. van Oostenbrugge, Martin P. J. van Boxtel, Frans R. J. Verhey, Sebastian Köhler

**Affiliations:** 1 Alzheimer Centre Limburg, School for Mental Health and Neuroscience, Maastricht University, Maastricht, the Netherlands; 2 Departamento de Psiquiatria, Hospital Alvaro Cunqueiro, Complexo Universitario de Vigo, Vigo, Spain; 3 Department of Neurology, Cardiovascular Research Institute Maastricht, Maastricht University Medical Center, Maastricht, the Netherlands; Nathan S Kline Institute, UNITED STATES

## Abstract

**Aims/Hypothesis:**

Accumulating evidence suggests an association between coronary heart disease and risk for cognitive impairment or dementia, but no study has systematically reviewed this association. Therefore, we summarized the available evidence on the association between coronary heart disease and risk for cognitive impairment or dementia.

**Methods:**

Medline, Embase, PsycINFO, and CINAHL were searched for all publications until 8^th^ January 2016. Articles were included if they fulfilled the inclusion criteria: (1) myocardial infarction, angina pectoris or coronary heart disease (combination of both) as predictor variable; (2) cognition, cognitive impairment or dementia as outcome; (3) population-based study; (4) prospective (≥1 year follow-up), cross-sectional or case-control study design; (5) ≥100 participants; and (6) aged ≥45 years. Reference lists of publications and secondary literature were hand-searched for possible missing articles. Two reviewers independently screened all abstracts and extracted information from potential relevant full-text articles using a standardized data collection form. Study quality was assessed with the Newcastle-Ottawa Scale. We pooled estimates from the most fully adjusted model using random-effects meta-analysis.

**Results:**

We identified 6,132 abstracts, of which 24 studies were included. A meta-analysis of 10 prospective cohort studies showed that coronary heart disease was associated with increased risk of cognitive impairment or dementia (OR = 1.45, 95%CI = 1.21–1.74, p<0.001). Between-study heterogeneity was low (*I*^2^ = 25.7%, 95%CI = 0–64, p = 0.207). Similar significant associations were found in separate meta-analyses of prospective cohort studies for the individual predictors (myocardial infarction, angina pectoris). In contrast, meta-analyses of cross-sectional and case-control studies were inconclusive.

**Conclusion/Interpretation:**

This meta-analysis suggests that coronary heart disease is prospectively associated with increased odds of developing cognitive impairment or dementia. Given the projected worldwide increase in the number of people affected by coronary heart disease and dementia, insight into causal mechanisms or common pathways underlying the heart-brain connection is needed.

## Introduction

Coronary heart disease (CHD) is the leading cause of death worldwide [1]. An estimated 7.4 million people died from CHD in 2012 [2]. CHD is a broad disease category and consists of several conditions with myocardial infarction (MI) and angina pectoris (AP) being the most prevalent ones. CHD affects the vascular system supplying the heart muscle due to build-up of atheromatous plaques that cover the lining of the coronary arteries [3].

At the same time, dementia is an important health problem due to increasing incidence rates and its impact on health and daily life [4]. Major modifiable risk factors for cognitive impairment and dementia relate to or impact the vascular system including hypertension, smoking, obesity, diabetes, hypercholesterolemia and lack of physical exercise [5, 6]. Notably, these factors are also risk factors for CHD [7]. While CHD is a candidate risk factor for dementia or cognitive impairment, the evidence base has not been established to a similar extent, yet [8]. In a recent systematic review of the literature on modifiable risk factors, several studies on heart disease were identified, of which the majority reported a higher risk for cognitive impairment or dementia [8]. Some other types of heart disease have been related to cognitive decline or dementia risk, too, with most substantial evidence for atrial fibrillation [9–11]. A meta-analysis of 7 prospective studies found that individuals with atrial fibrillation had a 36 percent increased risk of developing cognitive impairment or dementia [9]. To date, no meta-analysis exists for major heart diseases such as MI and AP.

Therefore, the aim of the present study is to summarize the outcome of all available population-based studies investigating the relation between CHD, notably MI, and AP, and risk for cognitive impairment or dementia in a systematic review and meta-analysis.

## Materials and methods

### Data sources and searches

The literature search was conducted in Medline, Embase, PsycINFO, and CINAHL. The search string consisted of predictor-related terms (e.g. myocardial infarction, angina pectoris), outcome-related terms (e.g. dementia, Alzheimer, cognition), as well as some specific limitations (e.g. only studies in human, language restrictions). The complete search strategy is provided in S1 Appendix.

### Study selection

All publications until 8^th^ January 2016 were included if they fulfilled the following eligibility criteria: 1) MI, AP, or a CHD variable that is a combination of MI and AP (e.g. ischemic heart disease (IHD)) as predictor variable; 2) cognition, cognitive impairment or dementia as outcome; 3) population-based study; 4) prospective (≥1 year follow-up), cross-sectional or case-control study design; 5) ≥100 participants; and 6) aged ≥45 years. Reference lists of publications and secondary literature (review articles, editorials, book chapters, etc.) were hand-searched for possible missing articles.

### Data extraction and quality assessment

The selection process followed the PRISMA (Preferred Reporting Items for Systematic reviews and Meta-Analyses) and MOOSE (Meta-analysis Of Observational Studies in Epidemiology) guidelines (S2 and S3 Appendices) [12, 13]. Titles and abstracts were screened by two independent assessors (KD, MMFR) based on the abovementioned eligibility criteria. Next, full text articles of potentially relevant citations were scrutinized by two independent investigators (KD, MMFR). A standardized data collection form was used to extract information such as study design, study cohort, demographics, predictor variable, outcome, and effect estimate. In case of discrepancy, discussion with a third reviewer (SK) took place. Corresponding authors were contacted by e-mail if full-text articles were not available or information was missing (e.g. effect estimates, sample sizes, definition of CHD) or ambiguous (with maximum three reminders in case of non-response). The Newcastle-Ottawa Scale (NOS) was used to asses study quality [14]. For cross-sectional studies, an adapted version of the NOS was applied (S4 Appendix).

### Data synthesis and analysis

Studies qualifying for pooling in meta-analyses were analyzed with random effects models to estimate the pooled odds ratios (OR) and their 95% confidence intervals (CI). Estimates from the most fully adjusted model were used. Meta-analyses were conducted for each exposure separately, i.e. for MI, AP, and CHD. The latter included all studies that reported a risk estimate for MI, AP or a combination of MI and AP. In case a study reported risk estimates for multiple exposures the combination estimate (first choice) or the effect estimate with the smallest standard error (i.e. largest sample size; second choice) was chosen. Studies with overlapping study populations were only included if they used other study designs (e.g. cross-sectional and prospective). Heterogeneity among studies was assessed using the *I*^2^ statistic and the 95% CI for *I*^2^ was calculated using the non-central χ^2^ approach. Potential sources of heterogeneity (including mean age at baseline, mean follow-up duration, percentage of women, outcome measurement and study quality) were explored by meta-regression. The 95% prediction interval was estimated for each meta-analysis including at least 3 observational studies. This measure takes into account the between-study heterogeneity and provides an interval for the expected estimate of a future observational study and has been recommended to be standardly included in meta-analysis [15]. Potential publication bias (i.e. small study effects) was assessed by visual inspection of funnel plots and Egger’s test. All tests were two-sided at an alpha-level of 0.05 and all analyses were done with Stata 13.1 (StataCorp, TX).

## Results

**Fig 1:**
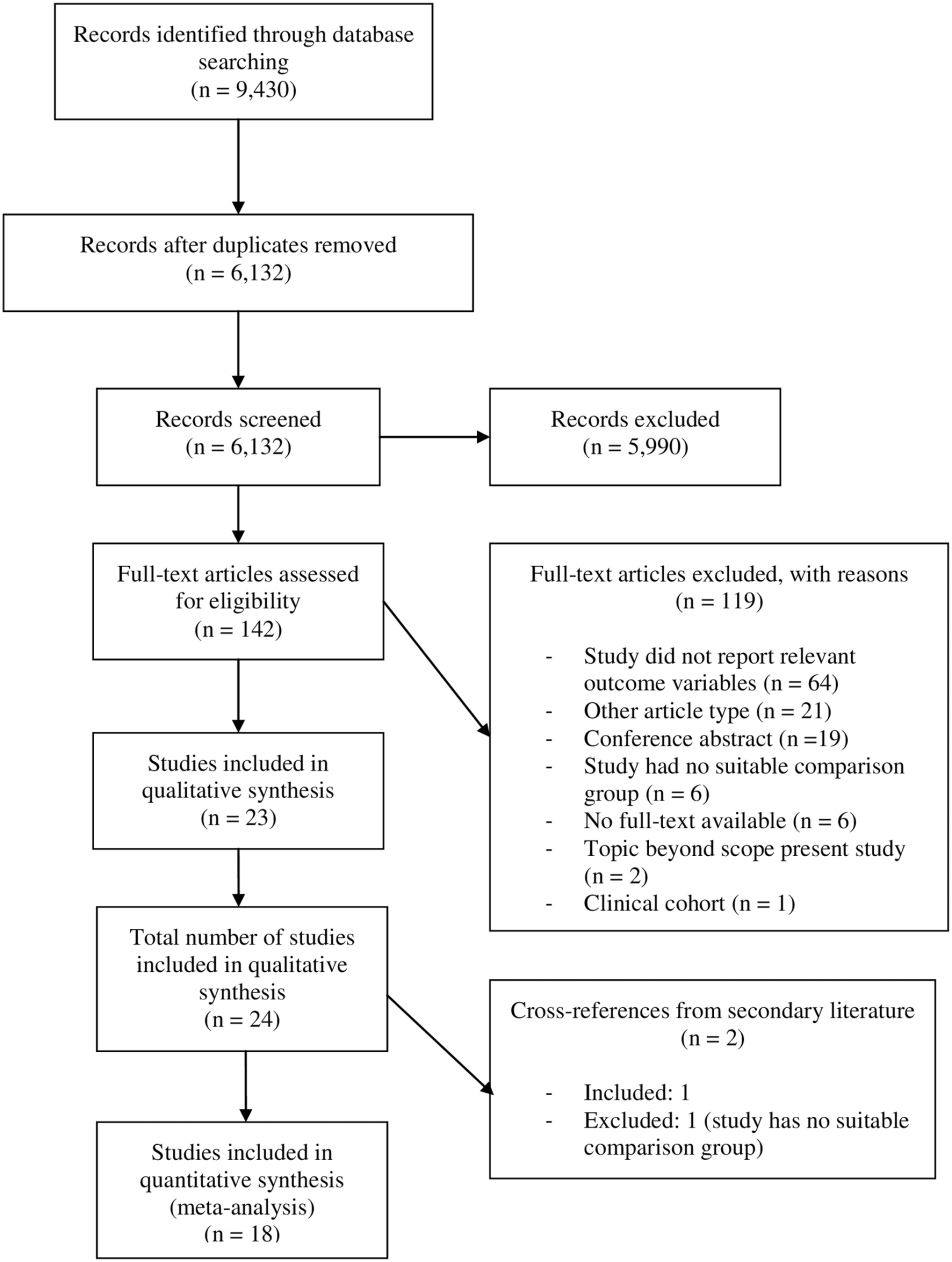
Flowchart of the literature search and selection.

The search yielded 6,132 abstracts, of which 142 (2.3%) were included for full-text review. Of these, 119 were excluded due to different reasons based on the exclusion criteria (Fig 1). Six authors were contacted to obtain full-text articles that were not available to us, of which 5 responded to our request. Additionally, 10 authors were contacted for missing or ambiguous information, of whom 7 responded. Two additional studies were found from cross-references [16, 17], of which one could be included [16]. This resulted in 8 cross-sectional studies, 5 case-control studies, 10 prospective cohort studies and 1 study with both cross-sectional and prospective analyses (designated as cross-sectional regarding study quality). Quality assessment of all 24 included studies was sufficient (overall mean NOS score = 6.8, SD = 1.29, range = 3–9). Separate analyses for each study design showed similar results for prospective (mean NOS score = 6.91, SD = 1.04, range = 5–8) and cross-sectional studies (mean NOS score = 7.22, SD = 0.97, range = 6–9), but the quality of case-control studies was somewhat lower (mean NOS score = 5.8, SD = 1.92, range = 3–8), mainly due to the effects of one particular study with a score of 3. All 24 studies and their details and results are summarized in detail in Tables 1–3 and Table A in S5 Appendix.

**Table 1:** Characteristics of prospective cohort studies assessing the relation between angina pectoris, myocardial infarction, coronary heart disease and cognition or dementia.

Authors	Cohort/ sample/age/ follow-up	Outcome/cognitive test, diagnostic criteria	Predictor/ ascertainment of exposure	Adjustment for confounders	Most important results
**Aronson et al., 1990**[18]	Bronx Aging study; N = 442; mean age: 79.2; FU range = 2–7 years	Dementia; annual exam measures (including cognitive tests), interview with proxy informant, EEG, CT or MRI, psychiatric assessment, assignment of an ischemic score, DSM-III criteria, NINCDS-ADRDA, neuropathological confirmation	MI; medical and laboratory studies (e.g. blood sample, ECG)	Sex, age, word fluency, Blessed IMC error score	Significant association between MI and dementia (HR = 1.8 (1.03–3.2))
**Kalmijn et al., 1996**[25]	Zuthpen Elderly study; N = 353; mean age: 74.6; 3-year FU	Cognitive decline; drop of >2 points on the MMSE	CHD; diagnosis of MI or AP (self-report verified by medical records, ECGs, hospital discharge data, and notes from GP)	Age, education, baseline MMSE score	No significant association between CHD and cognitive decline (OR = 1.7 (0.8–3.5))
**Ross et al., 1999**[26]	Honolulu-Asia Aging study; N = 2,916; age range: 71–93; maximum FU = 28 years	VaD; cognitive screening with CASI, additional cognitive testing, interview with proxy-informant, full-dementia examination (interview, neurological examination, neuropsychological test battery), brain CT, laboratory tests, DSM-III-R criteria, expert panel consensus diagnosis	CHD; diagnosis of MI or AP (medical history, ECG)	Age, education, hypertension, diabetes, Western diet preference, use of Vitamin E, 1-hour postprandial glucose at examination 1	Significant association between CHD and VaD (OR = 2.5 (1.35–4.62))
**Kivipelto et al., 2002**[16]	North Karelia Project and FINMONICA study; N = 1,287; age range: 65–79; mean FU = 21 years	AD, AD/VaD; 1) screening phase with MMSE; 2) clinical phase where participants (MMSE ≤ 24) underwent neurological, cardiovascular and neuropsychological examinations; 3) differential diagnosis phase (blood test, brain imaging, ECG and cerebrospinal fluid analysis) based on established criteria (DSM-IV, NINCDS-ADRDA)	MI; self-report of a physician diagnosis	Age, sex, education, smoking, alcohol consumption, APOE genotype	MI (as of the late-life visit) was significantly associated with AD (OR = 2.1 (1.1-4-5)) and AD or VaD (OR = 2.5 (1.2–5.4)). MI at midlife was not associated with AD.
**Verhaeghen et al., 2003** [27]	Berlin Aging Study; N = 206; mean age >70; FU = 4 years	Cognitive decline; perceptual speed (Digit Letter, Identical Pictures), episodic memory (Paired Associates, Memory for text), fluency (Categories, Word Beginnings), knowledge (Vocabulary, Spot-a-Word), intelligence (composite based on four separate composites)	CHD; typical angina, stenocardia, nitrate therapy, family doctor’s diagnosis, ECG abnormalities	Age, sex, SES, dementia status	CHD was not associated with cognitive decline
**Newman et al., 2005**[23]	Cardiovascular Health study; N = 2,539; median age: 74; mean FU = 5.4 years	Dementia, AD with or without VaD, AD with no VaD; annual measures of cognition, detailed neurological and neuropsychological examinations, medical records, physician questionnaires, proxy-informant interviews, brain MRI, expert panel consensus diagnosis, several diagnostic criteria (e.g. NINCDS-ADRDA)	MI, AP; self-report confirmed by medical records, test results (e.g. ECG), or medication use at study entry (e.g. nitroglycerin)	Age at baseline, education, race, income, APOE genotype, modified MMSE score at time of brain MRI	The incidence of dementia was higher in those with MI or AP. In adjusted models, these associations were no longer or borderline significant (e.g. dementia: HR = 1.3 (1.0–1.9))
**Hayden et al., 2006**[20]	Cache County study; N = 3,264; mean age: 74; mean FU = 3.2 years	Dementia, AD, VaD; multi-stage cognitive screening procedure (e.g. cognitive test, proxy-informant questionnaires), full clinical assessment (neurological and neuropsychological assessment, laboratory tests, brain-imaging, expert panel consensus diagnosis, several diagnostic criteria (DSM-III-R, NINCDS-ADRDA, NINDS-AIREN)	MI; self-report or proxy-informant-report of a physician diagnosis together with self-reported treatment	Age, sex, education, hypertension, high cholesterol, diabetes, obesity, stroke, CABG, APOE genotype	MI was not significantly associated with dementia (HR = 1.13 (0.59–2.03))
**Ikram et al., 2008**[21]	Rotterdam study; N = 5,578; mean age > 68; maximum FU = 15 years	Dementia; cognitive screening tests, CAMDEX, neuropsychological assessment, imaging data, record linkage, expert panel consensus diagnosis, several diagnostic criteria (DSM-III-R, NINCDS-ADRDA, NINDS-AIREN)	MI (recognized); based on Q-wave (self-reported MI confirmed by ECG abnormalities) and non-Q-wave MI (self-reported MI confirmed by only clinical data) Unrecognized MI; no self-reported or documented MI, but based on only ECG abnormalities	Age, sex, systolic blood pressure, diastolic blood pressure, BMI, atrial fibrillation, diabetes, current smoking, intima media thickness, total cholesterol, HDL-cholesterol, APOE genotype	Recognized MI was not significantly associated with dementia risk (HR = 1.12 (0.77–164)). Unrecognized MI was associated with an increased risk of dementia, but only in men (HR = 2.14 (1.37–3.35))
**Chen et al., 2011**[24]	Anhui cohort study; N = 1,307; mean age > 65; median FU = 3.9 years	Dementia; GMS-AGECAT diagnosis, death register (for cases who died in the FU before re-interviewing), psychiatrist’s diagnosis (for patients from case-control study)	AP; doctor’s diagnosis	Age, sex, education, main occupation, annual income, urban rurality, BMI, smoking habits, hobby’s (e.g. playing chess, pet), relationship with others, living with others, worrying, hypochondriasis, anything severely upsetting, horrifying experience	AP was significantly associated with incident dementia (OR = 2.58 (1.01–6.59))
**Haring et al., 2013**[19]	Women’s Health Initiative Memory study; N = 6,455; age range: 65–79; median FU = 8.4 years	Possible dementia, MCI, possible dementia or MCI; cognitive screening (3MSE), CERAD battery of neuropsychological tests and standardized interviews, interview with proxy-informant, review meeting with local physician (medical history, neuropsychiatric evaluation), brain CT, laboratory tests, expert panel consensus diagnosis, several diagnostic criteria (DSM-IV, CERAD)	MI; based on self-report or evolving Q-wave (ECG) AP; self-report	Age, education, race, HTR arm, baseline 3MSE, alcohol intake, smoking, physical activity, diabetes, sleep hours, hypertension, BMI, depression, waist-hip ratio, hypercholesterolemia, aspirin use	MI was significantly associated with for possible dementia or MCI (HR = 2.10 (1.40–3.15)) AP was moderately associated with possible dementia or MCI (HR = 1.45 (1.05–2.01))
**Lipnicki et al., 2013**[22]	Sydney Memory and Ageing study; N = 660*; mean age: 78.59; mean FU = 23 months, 12 days	Decline to MCI or dementia; MCI: participant or informant cognitive complaint, cognitive impairment on objective testing, no dementia diagnosis, normal function or minimal impairment in instrumental activities of daily living, expert panel consensus diagnosis, diagnostic criteria; dementia: expert panel consensus diagnosis, diagnostic criteria (DSM-IV)	MI; self-report of a physician diagnosis AP; doctor’s diagnosis CHD; combination of MI and AP	Age, sex	No significant associations between MI (OR = 1.12 (0.58–2.19)), AP (OR = 0.98 (0.51–1.88)) or CHD (OR = 0.97 (0.55–1.71)) ^a^

3MSE, Modified Mini-Mental State Examination; AD, Alzheimer’s disease; APOE, apolipoprotein E; AP, angina pectoris; Blessed IMC, Blessed Test of Information, Memory, and Concentration; BMI, body mass index; CABG, coronary artery bypass graft surgery; CAMDEX, Cambridge Examination for Mental Disorders in the Elderly; CASI, Cognitive Abilities Screening Instrument; CERAD, Consortium to Establish a Registry for Alzheimer’s Disease; CHD, coronary heart disease; CT, computer tomography; DSM-III, Diagnostic and Statistical Manual of Mental Disorders (third edition); DSM-IV, Diagnostic and Statistical Manual of Mental Disorders (fourth edition); ECG, electrocardiography; EEG, electroencephalography; FU, follow-up; GMS-AGECAT, Geriatric Mental State-Automated Geriatric Examination for Computer Assisted Taxonomy; GP, general practitioner; HDL, high-density lipoprotein; HR, hazard ratio; HTR-arm, Women’s Health Initiative Hormone Trial Randomization assignment; MCI, mild cognitive impairment; MI, myocardial infarction; MMSE, Mini-Mental State Examination; MRI, magnetic resonance imaging; HR, hazard ratio; NINCDS-ADRDA, National Institute of Neurological and Communicative Disorders and Strokes—Alzheimer's Disease and Related Disorders Association criteria; NINDS-AIREN, National Institute of Neurological Disorders and Strokes—Association International pour la Recherché l'enseignement en Neurosciences criteria; OR, odds ratio; VaD, vascular dementia.

^a^ Number of participants and ORs obtained after contact with corresponding author.

**Table 2:** Characteristics of case-control studies assessing the relation between angina pectoris, myocardial infarction, coronary heart disease and cognition or dementia.

Authors	Cohort/sample (cases and controls), age	Outcome/cognitive test, diagnostic criteria	Predictor/ ascertainment of exposure	Adjustment for confounders	Most important results
**Brayne et al., 1998**[31]	The Cambridge City over -75s Cohort study (CC75C); N = 376 (36 cases; 340 controls); mean age >77	Dementia, AD; CAMDEX interview	MI; self-report or proxy-informant-reported history of MI	Age, sex	History of MI associated with dementia risk (OR = 2.94 (1.2–7.21))
**Massaia et al., 2001**[29]	Persons visiting the Geriatric Institute of the University of Torino, Italy; N = 456 (228 cases; 228 controls); mean age > 74	AD; DSM-III and NINCDS-ADRDA criteria	MI; not described	Not applicable	No significant difference between cases and controls with regard to MI
**Bursi et al., 2006**[28]	Rochester Epidemiology Project; N = 1,832 (916 cases; 916 controls); median age cases: 82 years	Dementia; record linkage, screening of medical records, confirmation by neurologist, DSM-IV criteria	MI; record linkage, screening of medical records based on discharge diagnosis codes, validation of diagnosis based on standardized criteria	None	No significant association between MI and dementia (OR = 1.0 (0.62–1.62))
**Hughes et al., 2010**[32]	HARMONY study; N = 3,779 (355 cases; 3,424 controls); mean age: 79.81	Dementia, AD; telephonic cognitive screening, in-person clinical evaluation including neurological and neuropsychological examination, several diagnostic criteria, expert panel consensus diagnosis	AP; self-reported	Not applicable	No significant association between AP and dementia (OR = 0.86 (0.66–1.13)) or AD (OR = 0.80 (0.58–1.11))^a^
**Takahashi et al., 2012** [30]	Subjects living in Olmsted County, USA; N = 410 (205 cases; 205 controls); mean age: 81.9	VaD; medical history, neuroimaging studies, clinical diagnosis from medical records, NINDS-AIREN criteria	MI, AP; medical records including physician notes, laboratory data, letters, non-visit care information, hospitalizations and dismissal diagnoses	None	No significant association between dementia risk and MI (OR = 1.11 (0.66–1.87)) or AP (OR = 1.22 (0.79–1.88))

AD, Alzheimer’s disease; AP, angina pectoris; CAMDEX, Cambridge Examination for Mental Disorders in the Elderly; DSM-III, Diagnostic and Statistical Manual of Mental Disorders (third edition); DSM-IV, Diagnostic and Statistical Manual of Mental Disorders (fourth edition); MI, myocardial infarction; NINCDS-ADRDA, National Institute of Neurological and Communicative Disorders and Strokes—Alzheimer's Disease and Related Disorders Association criteria; NINDS-AIREN, National Institute of Neurological Disorders and Strokes—Association International pour la Recherché l'enseignement en Neurosciences criteria; OR, odds ratio; USA, United Stated of America; VaD, vascular dementia.

^a^ Crude OR calculated based on numbers reported in Table 1 of the article.

**Table 3:** Characteristics of cross-sectional studies assessing the relation between angina pectoris, myocardial infarction, coronary heart disease and cognition or dementia.

Authors	Cohort/sample/ age	Outcome/ cognitive test, diagnostic criteria	Predictor/ ascertainment of exposure	Adjustment for confounders	Most important results
**Breteler et al., 1994**[33]	Rotterdam study; N = 4,971; age range: 55–94	Cognitive function; MMSE	MI; ECG abnormalities reviewed by a cardiologist	Age, sex, education, smoking	History of MI was associated with lower cognitive scores
**Petrovitch et al., 1998**[35]	Honolulu-Asia Aging study; N = 341; mean age >77	Cognitive function; CASI (poor cognitive performance was defined as a score of < 74)	MI; diagnosis of MI (chest pain with ECG changes or cardiac enzyme elevation, temporal ECG changes considered to be diagnostic of interim MI) based on several sources (e.g. surveillance of all hospital discharge records, death certificates) and subjected to standardized review and classification by a consensus diagnosis committee	Age, years of education, and years of childhood spent in Japan	No significant association between MI and cognitive performance (OR = 1.3 (0.8–1,9)
**Elwood et al., 2002**[34]	Caerphilly study; N ≈ 1,500; age range: 55–69	Cognitive function; AH4-1 test, CAMCOG, MMSE and CRT	MI; questionnaire on vascular events, admission lists of local hospitals, hospital and GP notes, chest ECG AP; questionnaire on vascular events, admission lists of local hospitals, hospital and GP notes, chest ECG	Age, social class, (mood)	Significant associations between cognitive function and past MI or the presence of AP
**Singh-Manoux et al., 2003**[37]	Whitehall II study; N = 5,812; age range: 46–68	Cognitive function; memory test, AH4-1 test, Mill Hill Vocabulary test, phonemic and semantic fluency	MI, AP; validated diagnosis based on clinical test abnormality or physician confirmation CHD; validated MI or AP and doctor-diagnosed CHD	Age, employment grade, (hypertension, cholesterol, cigarette smoking)	MI, AP and CHD were associated with poor cognitive function
**Verhaeghen et al., 2003**[27]	Berlin Aging Study; N = 516; mean age >70	Cognitive function; perceptual speed (Digit Letter, Identical Pictures), episodic memory (Paired Associates, Memory for text), fluency (Categories, Word Beginnings), knowledge (Vocabulary, Spot-a-Word), intelligence (composite based on four separate composites)	MI; case history, interview with general physician, ECG abnormalities CHD; typical angina, stenocardia, nitrate therapy, family doctor’s diagnosis, ECG abnormalities	Age, sex, SES, dementia diagnosis	MI was negatively associated with fluency, knowledge and intelligence composite^a^ CHD was negatively associated with cognition
**Singh-Manoux et al., 2008**[40]	Whitehall II study; N = 5,837; mean age: 61.0	Cognitive function; memory test, AH4-1 test (reasoning), Mill Hill Vocabulary test, phonemic and semantic fluency, MMSE	CHD; non-fatal MI (questionnaire data, study and hospital ECGs, cardiac enzymes and physician records) and definite AP (self-report of symptoms corroborated by information from medical records for nitrate medication or abnormalities on ECG, exercise ECG or coronary angiogram)	Age, education, marital status, use of medication for cardiovascular disease	In both men and women, CHD was associated with lower cognitive scores on reasoning, vocabulary and the MMSE. In women, CHD was also associated with lower scores on phonemic and semantic fluency
**Roberts et al., 2010**[36]	Mayo Clinic Study of Ageing; N = 1,969; median age: 80.4	MCI; cognitive concern by a physician, patient, or nurse, impairment in ≥1 cognitive domains (executive function, memory, language visuospatial skills), essentially normal functional activities, no dementia diagnosis a-MCI: MCI with memory impairment na-MCI: MCI with no memory impairment	MI (definite); three sources: 1) self-report of a physician diagnosis; 2) ICD-codes based on information from the medical index of the Rochester Epidemiology Project; 3) validated diagnoses from a separate surveillance study AP (probable); two sources: 1) self-report of a physician diagnosis with or without self-report of treatment with nitrates, beta-blockers, or calcium channel blockers specifically stated as treatment for angina; 2) ICD-codes from the medical records-linkage system	Age, sex, and years of education, diabetes, hypertension, stroke, BMI, depression, dyslipidemia, APOE genotype	MI and AP were not significantly associated with MCI, a-MCI or na-MCI
**Arntzen et al., 2011**[38]	Tromsø study; N = 5,033; mean age: 58.8 (men)/ 58.2 (women)	Cognitive function; twelve word memory test, digit-symbol coding test, tapping test Cognitive impairment; lowest quintile on cognitive test scores	CHD; self-reported MI or AP	Age, education, physical activity, depression, current smoking, hypertension, hypercholesterolemia, low HDL-cholesterol, obesity, diabetes	No significant associations between CHD with any of the cognitive tests
**Heath et al., 2015**[39]	UK National Health Service; N = 616,245; age range: 40–64	Dementia; the presence ever of one of a specified set of GP codes for dementia or the prescription ever of an anticholinesterase inhibitor	IHD^b^; GP codes for MI or AP	Age, sex, SES, presence of neurodegenerative disorder or learning disability	Significant association between IHD and dementia (OR = 1.9 (1.5–2.4))

AH4-1, Alice Heim 4–1; a-MCI, amnestic mild cognitive impairment; AP, angina pectoris; APOE, apolipoprotein E; BMI, body mass index; CASI, Cognitive Abilities Screening Instrument; CAMCOG, Cambridge Cognitive Examination; CHD, coronary heart disease; CRT, Choice Reaction Time test; ECG, electrocardiography; GP, general practitioner; HDL, high-density lipoprotein; ICD, International Classification of Diseases; IHD, ischemic heart disease; MCI, mild cognitive impairment; MI, myocardial infarction; MMSE, Mini-Mental State Examination; na-MCI, non-amnestic mild cognitive impairment; OR, odds ratio; SES, socioeconomic status; UK, United Kingdom.

^a^ More specific results were obtained after contact with the corresponding author.

^b^ Definition of IHD was obtained after contact with the corresponding author.

### Prospective cohort studies

From the eleven prospective cohort studies, seven focused on MI [16, 18–23], four on AP [19, 22–24], and four studies on the CHD compound [22, 25–27]. Of those focusing on MI, four studies did not find an association with dementia, Alzheimer’s disease, vascular dementia or decline to mild cognitive impairment or dementia [20–23]. Three studies did find a significant association between MI and dementia [18], Alzheimer’s disease and Alzheimer’s disease/vascular dementia (but only for MI ascertained at the late-life visit)[16], and possible dementia/mild cognitive impairment [19]. Two out of the four AP studies did find that AP increased the risk of dementia or possible dementia/mild cognitive impairment [19, 24], whereas the other two studies did not find an association [22, 23]. For the CHD compound, three studies did not find a relation with cognitive decline or decline to dementia/mild cognitive impairment [22, 25, 27], whereas one study found that CHD was a significant predictor of vascular dementia [26].

**Fig 2:**
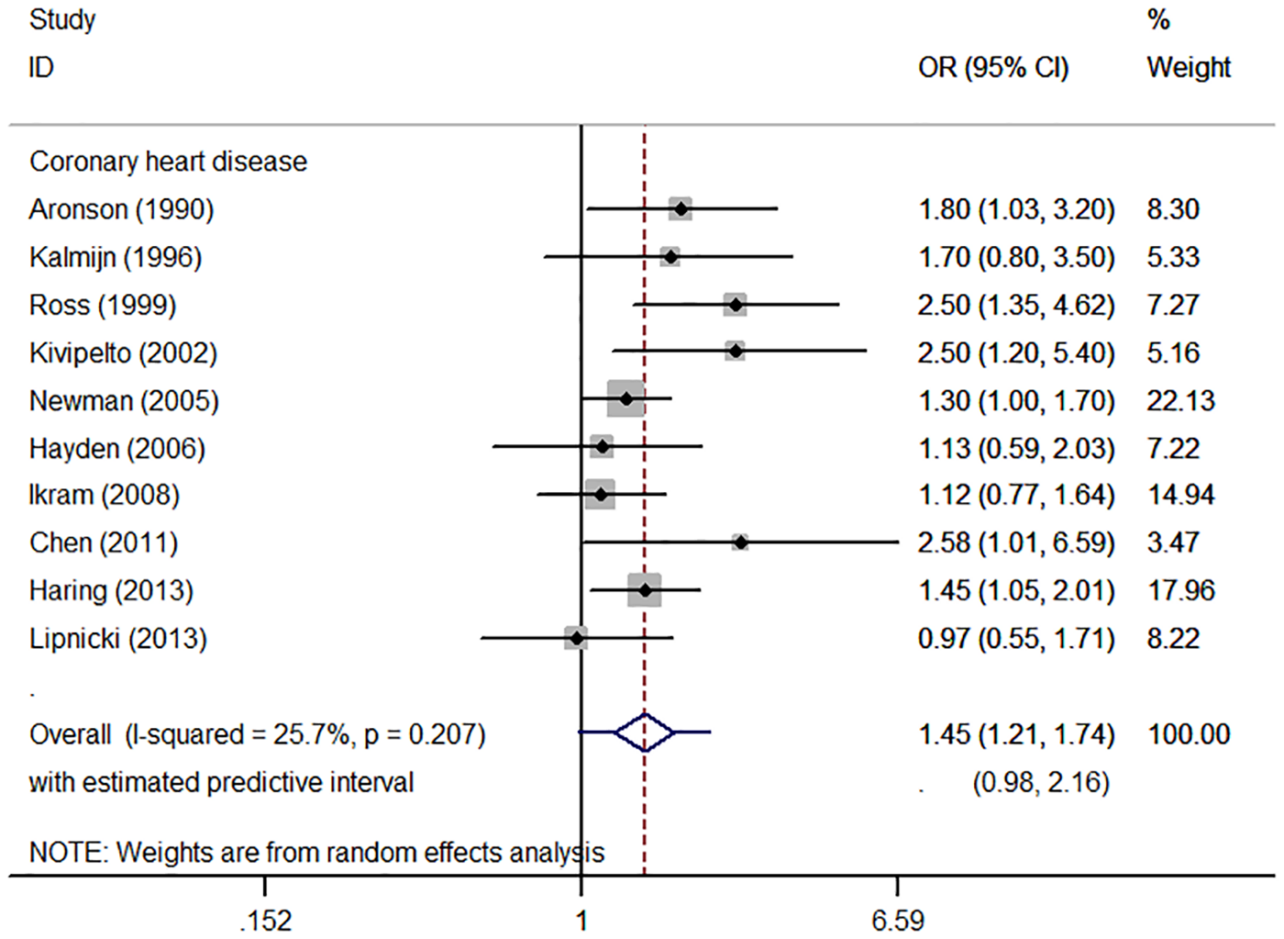
Forest plot of prospective cohort studies assessing the relation between coronary heart disease and cognitive impairment or dementia.

In the meta-analysis, a total of ten studies representing 24,801 persons could be included [16, 18–26]. CHD was associated with a 45% increased risk of dementia, cognitive impairment or cognitive decline (OR = 1.45, 95%CI = 1.21–1.74, p<0.001; Fig 2). Heterogeneity was low (*I*^2^ = 25.7%, 95%CI = 0–64, p = 0.207), without suggestion of small-study effects (Egger’s test, p = 0.739; Figure A in S5 Appendix). No statistically significant source of heterogeneity was identified in a meta-regression analysis. Associations were slightly stronger in studies (n = 7) focusing on dementia (OR = 1.55, 95%CI = 1.20–2.00, p = 0.001; *I*^2^ = 40.6%, 95%CI = 0–74, p = 0.121) [16, 18, 20, 21, 23, 24, 26]. There were too few studies to conduct separate meta-analyses for the different subtypes of dementia. Similar significant results were found for MI (OR = 1.46, 95%CI = 1.16–1.84, p = 0.001, Figure B in S5 Appendix) and AP (OR = 1.36, 95%CI = 1.12–1.65, p = 0.002, Figure C in S5 Appendix) separately.

### Case-control studies

Four out of five case-control studies reported on MI. Three of these found no association between MI and dementia or Alzheimer’s disease [28–30], whereas one nested case-control study did find a significant association between MI and dementia risk [31]. Two case-control studies investigated the relation between AP and risk for dementia, Alzheimer’s disease or vascular dementia. Both studies showed no significant associations [30, 32].

Four studies representing 6,397 individuals could be included in the meta-analysis [28, 30–32]. CHD was not significantly associated with risk of total or vascular dementia (OR = 1.14, 95%CI = 0.79–1.64, p = 0.482; Figure D in S5 Appendix). There were signs of moderate heterogeneity (*I*^2^ = 60.3%, 95%CI = 0–85, p = 0.056). There was no strong evidence for small-study effects based on the Egger’s test (p = 0.062) and visual inspection of the funnel plot (Figure E in S5 Appendix). No statistically significant source of heterogeneity was identified in a meta-regression analysis. Separate meta-analyses for MI and AP showed comparable non-significant results (MI: OR = 1.32, 95%CI = 0.78–2.21, p = 0.302, Figure F in S5 Appendix; AP: OR = 0.98, 95%CI = 0.71–1.36, p = 0.911, Figure G in S5 Appendix).

### Cross-sectional studies

Out of nine cross-sectional studies, six studies reported on MI [27, 33–37], three on AP [34, 36, 37], and five on the CHD compound (MI+AP) [27, 37–40]. Of the six studies investigating MI, four found a significant relation with poor cognitive functioning [27, 33, 34, 37], and two studies found no association with prevalent cognitive impairment [35, 36]. For AP, two studies found a significant association with poor cognitive functioning [34, 37], whereas one study found no association with mild cognitive impairment [36]. For the CHD compound studies, three studies found a significant association with poor cognitive functioning [27, 37, 40], one study found no relation with cognitive function or cognitive impairment [38], and one study found a significant association with dementia risk [39].

In the meta-analysis, four studies representing 623,588 persons could be included [35, 36, 38, 39]. CHD was not significantly associated with an increased risk of cognitive impairment or dementia (OR = 1.23, 95%CI = 0.76–1.97, p = 0.398; Figure H in S5 Appendix). In the CHD meta-analysis, substantial heterogeneity was observed (*I*^2^ = 81.2%, 95%CI = 26–91, p = 0.001). No statistically significant source of heterogeneity was identified in a meta-regression analysis, although inclusion of some study characteristics (e.g. mean age at baseline, outcome measurement and study quality) led to a reduction in *I*^2^ (e.g. mean age at baseline: 81.2% to 53.1%). There was no evidence for small-study effects based on the Egger’s test (p = 0.407) and visual inspection of the funnel plot (Figure I in S5 Appendix). Similar non-significant results were found for MI (OR = 1.11, 95%CI = 0.79–1.57, p = 0.548; Figure J in S5 Appendix). It was not possible to perform a meta-analysis for AP since there was only one study [36].

## Discussion

The results of the meta-analysis of prospective cohort studies indicate that individuals with CHD have, on average, a 45% increased risk of cognitive impairment or dementia. Separate meta-analyses of prospective cohort studies for the individual predictors (MI, AP) showed similar significant results. In contrast, meta-analyses of cross-sectional and case-control studies yielded no significant results, possibly due to the low number of studies included within these analyses and the moderate to substantial heterogeneity among studies. It has to be noted that, for cross-sectional studies, those studies that could not be included in the meta-analysis (those using different continuous outcome measures of cognitive functioning), majorly found lower cognitive abilities in CHD. The literature on CHD is mixed in general, with the majority of prospective and cross-sectional studies demonstrating a significant association with cognition or dementia, and most of the case-control studies showed no association.

The exact biological mechanism by which CHD is related to risk of cognitive impairment or dementia is still unknown, but several candidate pathways exist. CHD and dementia share common risk factors such as obesity, type-2 diabetes, smoking, hypertension, physical inactivity, and hypercholesterolemia [7, 8]. Post-hoc meta-regression analyses showed that there were no differences between studies (n = 3) that corrected for cardiovascular risk factors (diabetes, hypertension, high cholesterol) and studies that did not correct for these factors. In other words, the association between CHD and dementia risk cannot be solely explained by shared cardiovascular risk factors. Additionally, CHD can be associated with cardiac complications (atrial fibrillation, heart failure), whose association with cognitive impairment or dementia is well-established [7, 9]. Additionally, CHD and accompanying vascular insufficiency can lead to cerebrovascular changes such as reduced cerebral blood flow (which can lead to hypoperfusion) [41], white matter lesions and brain infarctions [21], which in turn are associated with reduced cognitive functioning and risk of dementia [42, 43]. CHD might however not itself be causally related to cognition, but brain-effects (e.g. cognitive impairment with vascular origin) might be due to underlying atherosclerosis, which increases both the risk of CHD and dementia [44, 45].

Policy makers and health workers must become more aware that identification of individuals at high risk for CHD or dementia is essential to intervene at an early stage by targeting shared modifiable risk factors (e.g. obesity, hypercholesterolemia, physical inactivity, hypertension, smoking).

Studies have shown that targeting these modifiable risk factors can be effective in reducing incidence rates and disease burden [5, 46]. Concerted actions focusing on the heart-brain connection might be key to fostering healthy aging. Future public health campaigns focusing on preventing CHD are dementia should join forces and consider placing a greater emphasis on targeting shared risk factors.

The strengths of this study include the use of large population-based studies with different study designs and the use of risk estimates that were pre-adjusted for confounding variables. Nevertheless, a number of limitations have to be mentioned. First, some studies based the ascertainment of the predictors on self-report or proxy-report, which can be prone to recall bias and underreporting, particularly given the relative older age of the included cohorts. This is particularly problematic in case-control studies, in which differential reporting bias may lead to exposure misclassification and diluted or even biased estimates. Fortunately, the majority of the included studies used validated or combined (e.g. self-report verified by validated) measurements to establish the exposure status. Related to this, is the underreporting of CHD events whereby stronger association might be distorted. This particularly applies to AP since AP is often missed, especially in comorbidity with atrial fibrillation. However, as shown by the separate meta-analyses of prospective cohort studies for MI and AP, there were no large differences between the different exposures. Second, substantial heterogeneity was observed in both cross-sectional and case-control studies. This can be related to differences in methodology across studies (e.g. assessment of dementia or cognitive functioning, ascertainment of exposure, variation between cohorts (e.g. gender specific) selection of study participants, follow-up duration and adjustment for important covariates). While meta-regression analyses did not identify any statistically significant source of heterogeneity (e.g. mean age at baseline, outcome measurement, follow-up duration), other methodological differences not included in the analyses might explain the between-study difference in effect estimates. By using a random-effects meta-analysis we have tried to account for variability within and between studies. The abovementioned issues related to cross-sectional and case-control studies might have led to the inconsistent findings between study designs. As prospective cohort studies are generally considered superior study designs to test the association between CHD exposure and dementia risk, we based our conclusion mainly on the results prospective cohort studies, but thereby not ignoring the findings of the other study designs. Third, the observed effects could probably be attributed to residual confounding in the original studies, although we used the most fully adjusted models. Fourth, studies were excluded if their CHD exposure was not a combination of purely MI and AP. For instance, studies reporting on IHD based on the the International Classification of Diseases and Related Health Problems (ICD-10) codes for IHD (I20-I25) were excluded because some of the codes also include coronary atherosclerosis and coronary artery aneurysm which are more causes of IHD [47]. While this has led to the exclusion of studies affirming the association between CHD and cognitive impairment or dementia, our focus was on MI and AP as the most prevalent conditions. Using a broader search strategy that includes all causes of IHD led to more than 12,000 search hits (now 6,132), which was considered unfeasible. Fifth, there were unfortunately too few studies to conduct separate meta-analyses for the different subtypes of dementia. Sixth, it would have been interesting to conduct stratified meta-analyses for gender, but unfortunately gender-specific risk estimates are scarce.

In conclusion, CHD was associated with an increased risk of cognitive impairment or dementia in prospective cohort studies. More mechanistic studies are needed that focus on the underlying biological pathways (e.g. left ventricular dysfunction, cerebral small vessel disease,hypoperfusion) and shared risks (e.g. hypertension, arterial stiffness, common genetic variants) that link CHD and risk of cognitive impairment or dementia.

## Supporting information

S1 Appendix Complete search strategy. (DOCX)

S2 Appendix MOOSE checklist. (DOCX)

S3 Appendix PRISMA checklist. (DOC)

S4 Appendix Newcastle-Ottawa quality assessment scale adapted for cross-sectional studies. (DOCX)

S5 Appendix Further supporting information. (DOCX)
