# Supplementary material for: Coronary heart disease and risk for cognitive impairment or dementia: Systematic review and meta-analysis
Source: PLoS One. 2017 Sep 8;12(9):e0184244. doi: 10.1371/journal.pone.0184244 (PMC5590905; doi:10.1371/journal.pone.0184244)
Supplement: S1 Appendix — (DOCX) [file pone.0184244.s001.docx]

**S1 Appendix. Complete search strategy.**

**Medline**

S1: (dement* or Alzheimer* or cognit* or neuropsychol*).af.

S2: (((ischemic or ischaemic or coronary) and (heart OR artery)) or myocardium or myocardial or angina).af.

S3: 1 and 2

S4: limit 3 to (humans and (dutch or english or french or german))

**Embase**

S1: (dement* or Alzheimer* or cognit* or neuropsychol*).ti,ab.

S2: (((ischemic or ischaemic or coronary) and (heart or artery)) or myocardium or myocardial or angina).ti,ab.

S3: 1 and 2

S4: limit 3 to (humans and (dutch or english or French or german))

**PsycINFO**

S1: dement# OR Alzheimer OR cognit# OR neuropsychol#

S2: (((ischaemic OR ischemic OR coronary) AND (heart OR artery)) OR myocardium OR myocardial OR angina)

S3: S1 AND S2

**CINAHL**

S1: (((ischaemic OR ischemic OR coronary) AND (heart OR artery)) OR myocardium OR myocardial OR angina)

S2: dement# OR Alzheimer OR cognit# OR neuropsychol#

S3: S1 and S2
