# Supplementary material for: Coronary heart disease and risk for cognitive impairment or dementia: Systematic review and meta-analysis
Source: PLoS One. 2017 Sep 8;12(9):e0184244. doi: 10.1371/journal.pone.0184244 (PMC5590905; doi:10.1371/journal.pone.0184244)
Supplement: S4 Appendix — (DOCX) [file pone.0184244.s004.docx]

**S4 Appendix. Newcastle-Ottawa quality assessment scale adapted for cross-sectional studies.**

Note: A study can be awarded a maximum of one star for each numbered item within the Selection and Outcome categories, with the exception for ‘Assessment for outcome’ from the Outcome category. A maximum of two stars can be given for Comparability.

**Selection** (Maximum 4 stars)

1. Representativeness of the exposed cohort

a) Truly representative of the general population (random sampling) *****

b) Somewhat representative of general population (non-random sampling) *****

c) Selected group of users (e.g. nurses, volunteers)

d) No description of the sampling strategy

1. Sample size, response rate, and comparability between respondent and non-respondents

a) Sample size is justified, response rate AND the comparability between respondents and non-respondents characteristics are described *****

b) Sample size is justified and the response rate OR the comparability between respondents and non-respondents characteristics is described *****

c) Sample size is justified, but no description of the response rate and the characteristics of the responders and non-responders

d) Sample size is not justified, and there is no description of the response rate or the characteristics of the responders and non-responders

1. Selection of the non-exposed cohort

a) Drawn from the same community as the exposed cohort *****

b) Drawn from a different source

c) No description of the derivation of the non-exposed cohort

1. 3) Ascertainment of exposure

a) Validated measurement tool *****

b) Non-validated measurement tool, but the tool is available or described *****

c) Self-report

d) No description

**Comparability** (Maximum 2 stars)

1. Comparability of cohorts on the basis of the design or analysis

a) Study controls for the most important factor *****

b) Study controls for any additional factor *****

c) No control for any important factor

**Outcome** (Maximum 3 stars)

1. Assessment of outcome

a) Independent blind assessment ******

b) Record linkage ******

c) Self-report

d) No description

1. Statistical test

a) The statistical test used to analyze the data is clearly described and appropriate, and the measurement of the association is presented, including confidence intervals and the probability level (p value) *****

b) The statistical test is not appropriate, not described or incomplete
