# Supplementary material for: Coronary heart disease and risk for cognitive impairment or dementia: Systematic review and meta-analysis
Source: PLoS One. 2017 Sep 8;12(9):e0184244. doi: 10.1371/journal.pone.0184244 (PMC5590905; doi:10.1371/journal.pone.0184244)
Supplement: S5 Appendix — (DOCX) [file pone.0184244.s005.docx]

**S5 Appendix. Further supporting information.**

- **S5 Table A.** Overview of studies assessing the relation between angina pectoris, myocardial infarction, coronary heart disease and cognition or dementia
- **S5 Figure A.** Funnel plot of prospective cohort studies included in the coronary heart disease meta-analysis showing the effect estimates by their standard errors.
- **S5 Figure B.** Forest plot of prospective cohort studies assessing the relation between myocardial infarction and cognitive impairment or dementia.
- **S5 Figure C.** Forest plot of prospective cohort studies assessing the relation between angina pectoris and cognitive impairment or dementia.
- **S5 Figure D.** Forest plot of case-control studies assessing the relation between coronary heart disease and cognitive impairment or dementia.
- **S5 Figure E.** Funnel plot of case-control studies included in the coronary heart disease meta-analysis showing the effect estimates by their standard errors.
- **S5 Figure F.** Forest plot of case-control studies assessing the relation between myocardial infarction and cognitive impairment or dementia.
- **S5 Figure G.** Forest plot of case-control studies assessing the relation between angina pectoris and cognitive impairment or dementia.
- **S5 Figure H.** Forest plot of cross-sectional studies assessing the relation between coronary heart disease and cognitive impairment or dementia.
- **S5 Figure I.** Funnel plot of cross-sectional studies included in the coronary heart disease meta-analysis showing the effect estimates by their standard errors.
- **S5 Figure J.** Forest plot of cross-sectional studies assessing the relation between myocardial infarction and cognitive impairment or dementia.

**S5 Table A. Overview of studies assessing the relation between angina pectoris, myocardial infarction, coronary heart disease and cognition or dementia.**

| **Authors** | **Study design** | **Predictor** | **Outcome** | **Included in meta-analysis? (If yes, which meta-analysis? If no, reason of exclusion?)** |
| --- | --- | --- | --- | --- |
| **Aronson et al., 1990** | Prospective | MI | Dementia | Yes, MI and CHD meta-analysis |
| **Kalmijn et al., 1996** | Prospective | CHD | Cognitive decline | Yes, CHD meta-analysis |
| **Ross et al., 1999** | Prospective | CHD | VaD | Yes, CHD meta-analysis |
| **Kivipelto et al., 2002** | Prospective | MI | AD, AD/VaD | Yes, MI and CHD meta-analysis |
| **Newman et al., 2005** | Prospective | MI, AP | Dementia, AD with no VaD, AD with or without VaD | Yes, MI, AP and CHD meta-analysis |
| **Hayden et al., 2006** | Prospective | MI | Dementia, AD, VaD | Yes, MI and CHD meta-analysis |
| **Ikram et al., 2008** | Prospective | MI | Dementia | Yes, MI and CHD meta-analysis^a^ |
| **Chen et al., 2011** | Prospective | AP | Dementia | Yes, AP and CHD meta-analysis |
| **Haring et al., 2013** | Prospective | MI, AP | Possible dementia, MCI, Possible dementia/MCI | Yes, MI, AP and CHD meta-analysis |
| **Lipnicki et al., 2013** | Prospective | MI, AP, CHD | Decline to MCI or dementia | Yes, MI, AP and CHD meta-analysis |
| **Brayne et al., 1998** | Nested case-control | MI | Dementia, AD | Yes, MI and CHD meta-analysis |
| **Massaia et al., 2001** | Case-control | MI | AD | No, only continuous outcome measures |
| **Bursi et al., 2006** | Case-control | MI | Dementia | Yes, MI and CHD meta-analysis |
| **Hughes et al., 2010** | Case-control | AP | Dementia, AD | Yes, AP and CHD meta-analysis |
| **Takahashi et al., 2012** | Case-control | MI, AP | VaD | Yes, MI, AP and CHD meta-analysis |
| **Elwood et al., 2002** | Cross-sectional | MI, AP | Cognitive function | No, only continuous outcome measures |
| **Singh-Manoux et al., 2003** | Cross-sectional | MI, AP, CHD | Cognitive function | No, only continuous outcome measures |
| **Verhaeghen et al., 2003** | Cross-sectional, prospective | MI, CHD | Cognitive function, cognitive decline | No, only continuous outcome measures |
| **Singh-Manoux et al., 2008** | Cross-sectional | CHD | Cognitive function | No, only continuous outcome measures |
| **Roberts et al., 2010** | Cross-sectional | MI, AP | MCI | Yes, MI and CHD meta-analysis |
| **Arntzen et al., 2011** | Cross-sectional | CHD | Cognitive function; cognitive impairment | Yes, CHD meta-analysis^b^ |
| **Heath et al., 2015** | Cross-sectional | CHD | Dementia | Yes, CHD meta-analysis |

AD, Alzheimer’s disease; AP, angina pectoris; CHD, coronary heart disease; MCI, mild cognitive impairment; MI, myocardial infarction; VaD, vascular dementia.

^a^ Hazard ratios for recognized myocardial infarction were taken into account due to larger number of participants

^b^ Odds ratios for women and the Tapping test were taken into account due to larger number of participants

**S5 Figure A. Funnel plot of prospective cohort studies included in the coronary heart disease meta-analysis showing the effect estimates by their standard errors.**

**S5 Figure B. Forest plot of prospective cohort studies assessing the relation between myocardial infarction and cognitive impairment or dementia.**

**S5 Figure C. Forest plot of prospective cohort studies assessing the relation between angina pectoris and cognitive impairment or dementia.**

**S5 Figure D. Forest plot of case-control studies assessing the relation between coronary heart disease and cognitive impairment or dementia.**

**S5 Figure E. Funnel plot of case-control studies included in the coronary heart disease meta-analysis showing the effect estimates by their standard errors.**

**S5 Figure F. Forest plot of case-control studies assessing the relation between myocardial infarction and cognitive impairment or dementia.**

**S5 Figure G. Forest plot of case-control studies assessing the relation between angina pectoris and cognitive impairment or dementia.**

**S5 Figure H. Forest plot of cross-sectional studies assessing the relation between coronary heart disease and cognitive impairment or dementia.**

**S5 Figure I. Funnel plot of cross-sectional studies included in the coronary heart disease meta-analysis showing the effect estimates by their standard errors.**

**S5 Figure J. Forest plot of cross-sectional studies assessing the relation between myocardial infarction and cognitive impairment or dementia.**
